# Effects of Passive- and Active-Modified Atmosphere Packaging on Physio-Chemical and Quality Attributes of Fresh In-Hull Pistachios (*Pistacia vera* L. cv. Badami)

**DOI:** 10.3390/foods8110564

**Published:** 2019-11-09

**Authors:** Abdollatif Sheikhi, Seyed Hossein Mirdehghan, Hamid Reza Karimi, Louise Ferguson

**Affiliations:** 1 Department of Horticultural Sciences, College of Agriculture, Vali-e-Asr University of Rafsanjan, Rafsanjan 7718897111, Kerman, Iran; abdollatif@ucdavis.edu (A.S.); hrkarimi2017@gmail.com (H.R.K.); 2 Department of Plant Sciences, University of California, Davis, CA 95616, USA

**Keywords:** active-modified atmosphere packaging, antioxidant enzyme, anthocyanin, microbial counts, fresh pistachio, postharvest quality

## Abstract

The effects of passive- and active-modified atmosphere packaging (passive- and active-MAP) were investigated on the physio-chemical and quality attributes of fresh in-hull pistachios stored at 4 ± 1 °C and 90 ± 5% R.H. Fresh pistachios were packaged under each of the following gas combinations: active-MAP1 (AMA1) (5% O_2_ + 5% CO_2_), AMA2 (5% O_2_ + 25% CO_2_), AMA3 (5% O_2_ + 45% CO_2_), AMA4 (2.5% O_2_ + 5% CO_2_), AMA5 (2.5% O_2_ + 25% CO_2_), and AMA6 (2.5% O_2_ + 45% CO_2_), all balanced with N_2_, as well as passive-MAP (PMA) with ambient air (21% O_2_ + 0.03% CO_2_ + 78% N_2_). Changes in quality parameters were evaluated after 0, 15, 30 and 45 days of storage. Results demonstrated that AMA6 and PMA had significantly lower (7.96 Log CFU g^−1^) and higher (9.81 Log CFU g^−1^) aerobic mesophilic bacteria counts than the other treatments. However, the AMA6 treatment decreased, kernel chlorophyll and carotenoid content, hull antioxidant capacity, and anthocyanin content. The PMA treatment produced a significant weight loss, 0.18%, relative to the other treatments. The active-MAP treatments were more effective than the passive-MAP in decreasing weight loss, microbial counts, kernel total chlorophyll (Kernel TCL), and kernel carotenoid content (Kernel CAC). The postharvest quality of fresh in-hull pistachios was maintained best by the AMA3 (5% O_2_ + 45% CO_2_ + 50% N_2_) treatment.

## 1. Introduction

The pistachio tree (*Pistacia vera* L.) is a diploid (2*n* = 30) member of the Anacardiaceae family. Pistachios are highly nutritious, rich in the phytochemical antioxidants; carotenoids (lutein), anthocyanin (cyanidin-3-galactoside, cyanidin-3-glucoside), flavonoids, vitamin E (α-tocopherol, γ-tocopherol), trans-resveratrol, phytosterols, and phytostanols [1,2,3]. The quantity of these health-promoting phytochemicals can be affected by both preharvest and postharvest factors; genetics (cultivar), cultivation practices, roasting, and processing. For example, sun drying pistachios decreases anthocyanins and vitamin E by 60% and 38%, respectively. Total phenolics, flavonoids, and stilbenes were significantly reduced after three days of sun exposure [4]. Increasingly, pistachios are being marketed as fresh. However, marketing fresh pistachios requires postharvest modified atmosphere packaging (MAP) and controlled atmosphere (CA) storage.

Modified atmosphere packaging, actively or passively, reduces oxygen (O_2_) and/or elevates carbon dioxide (CO_2_) concentrations [5,6,7]. Passive-MAP relies on the respiration rate of the produce and film permeability to produce its effects. Active-MAP is achieved by flushing packages with a known gas mixture, creating an atmosphere that evolves as a function of storage conditions, specifically the produce respiration rate and the film permeability [8]. Despite the successful application of passive- and active-MAP for a wide range of fruits and vegetables, such as in-hull walnuts [9], hazelnuts [10], almond kernels [11], pomegranates [12], cherry tomatoes [13], bell peppers [14], and watercress [15]. Ozturk et al. [16] reported passive-MAP could preserve fresh in-hull pistachio quality relative to vacuum and conventional packaging. There has been little investigation of the effects of MAP on fresh in-hull pistachios.

Although the popularity and consumption of fresh pistachios has increased sharply due to their health benefits and grower returns, the postharvest transport and marketing has remained primitive. Fresh pistachios are marketed without packaging on open trays at ambient temperatures, often outside. These conditions decrease postharvest life through hull and kernel degradation, weight loss, and secondary pathogenic infestation. The objectives of this study were to optimize a new packaging system, using initial flushing of O_2_, CO_2_, and N_2_ gases, for retail marketing of fresh in-hull pistachio fruits, and to understand physio-chemical changes in response to passive- and active-MAP.

## 2. Materials and Methods

### 2.1. Fruit Material

Commercially mature fresh in-hull pistachio (*Pistacia vera* L. cv. Badami) fruits were hand-harvested from a commercial orchard in Rafsanjan, Iran, transported within 2 h to overnight storage at 4 ± 1 °C before separating the nuts from the cluster and selecting for uniformity in size, shape, and color. Nuts with mechanical damage, sunburn, cracks, cuts and bruises, and obvious pathological infestation were discarded.

### 2.2. Experimental Design, Packaging, and Storage Conditions

The experiment was conducted as a factorial based on a completely randomized design (CRD) with four replications (Table 1). All parameters were measured at four storage times, 0, 15, 30, and 45 days after harvest. Weight loss which was not measured at day 0. Microbial counts were evaluated after 45 days as a completely randomized design, seven atmospheric treatments of four replications each. The packaging containers were impermeable polypropylene (PP) based plastic trays; 12 by 17 by 5 cm (total volume 1.02 L). The packages were filled with 250 ± 1 g of fresh in-hull pistachios, flushed with the seven treatment combinations by an industrial packaging machine equipped with a gas mixer linked to O_2_, CO_2_, and N_2_ (99.99% purity) cylinders. The packages were heat sealed with a polypropylene film with an O_2_ permeability of 1300 mL m^−2^ d^−1^ atm^−1^ and CO_2_ permeability of 7700 mL m^−2^ d^−1^ atm^−1^ at 23 °C. The 84 packages, four replications of seven treatment combinations, were evaluated at 15, 30, and 45 days, plus the four packages for the measurements at harvest, day 0.

### 2.3. Weight Loss, Firmness, and Microbial Counts

Weight loss was determined as percentage of the initial package weight. Firmness was determined separately for hulls and kernels using a digital pressure tester (model Lutron FG5020, Taiwan) fitted with an 8 mm flat-head probe for the hulls, and chisel probe for the kernels. Ten fruit for each replicate were randomly selected, and the results were expressed in Newtons (N). For microbial counts, a 10 g sample from each package was cut into pieces using sterile scissors, transferred to sterilized 100 mL borosilicate glass media bottles containing 90 mL of 1% peptone water (Merck, Darmstadt, Germany), and shaken for 15 min. Serial decimal dilutions were prepared using 1 mL of homogenized sample and 9.0 mL of 1% peptone water. Individual 0.2 mL aliquots were plated on petri dishes containing tryptic soy agar and incubated at 28 °C. The aerobic mesophilic bacteria colonies were counted (counts between 30 and 300 colonies) after 48 h. For mold and yeast counts, 0.2 mL of the dilutions was plated on potato dextrose agar (PDA) (Sigma–Aldrich) medium containing 0.1 g/L streptomycin and incubated for five days at 25 °C. Results were expressed as log of colony forming units per gram (Log CFU g^−1^) of fruit. Values were based on means of four replicates.

### 2.4. External Hull Color Evaluation

The fruit’s external hull (exocarp) color was measured using a chromameter (CR-400, Konica Minolta Inc., Osaka, Japan) equipped with a D65 illuminant source at an observation angle of 2°, which provided CIE L^*^, a^*^, and b^*^ values. The instrument was previously calibrated on a white plate standard (Y = 85.99, x = 0.3177, y = 0.3255). The measuring area of the chromameter was 8 mm in diameter, and the size of the pistachio nuts were at least 20 mm in length and 15 mm in width. The measured a^*^ and b^*^ values were used to calculate hue degree (*h^°^* = arctangent (b^*^/a^*^)), and chroma (C^*^ = (a^*2^ + b^*2^)^1/2^). Values were obtained randomly for 10 fruit per replicate and three measurements per fruit.

### 2.5. Total Chlorophylls and Carotenoids Content

To evaluate total chlorophylls (TCL) and carotenoids content (CAC), 1.0 g of hull and kernel were homogenized in 10 mL of acetone with 20% (*v*/*v*) water for 1 min at 13,000 rpm using an Ultra-Turrax homogenizer (Janke and Kunkel, Ika-Labortechnik, Germany) and centrifuged at 9072 g at 4 °C for 10 min. The absorbance was measured according to Lichtenthaler and Buschmann (2001) [17] at 663.2, 646.8, and 470 nm with a uv/vis spectrophotometer (Analytik Jena, Specord^®^ 250, Germany). Results were expressed as mg kg^−1^ of fresh weight.

### 2.6. Total Anthocyanins Content, Total Antioxidant Activity, and Total Phenolic Compounds

A 0.5 g of kernel skin (integument) sample was homogenized in 10 mL of 80% methanol containing 0.1% HCl for 1 min at 13,000 rpm in an Ultra-Turrax homogenizer and centrifuged at 9072 g at 4 °C for 10 min. The extract was used for quantifying total anthocyanins content (TAC) in the kernel’s skin (integument) using the colorimetric pH differential method according to Wrolstad (1976) [18]. The results were expressed as mg of cyanidin-3-galactoside (molar extinction coefficient = 30,200 L mol^−1^ cm^−1^) equivalent per kg of fresh weight. For the total antioxidant activity (TAA), 1 g of frozen sample (in liquid nitrogen and kept at −80 °C) of separated hulls and kernels was homogenized in an ice-water bath for 1 min at 13,000 rpm using an Ultra-Turrax homogenizer in 10 mL of 80% methanol containing 0.1% HCl, then centrifuged at 9072 g at 4 °C for 5 min. The TAA was determined using the method developed by Brand-Williams, Cuvelier, and Berset (1995), [19] using 2,2-diphenyl-1-picryl-hydrazyl (DPPH) as the free radical. For total phenolic compounds (TPC), 5 g of frozen sample (in liquid nitrogen and kept at −80 °C) of both hulls and kernels was homogenized separately in an ice-water bath for 1 min at 13,000 rpm using an Ultra-Turrax homogenizer in 10 mL of 50 mM phosphate buffer pH = 7.8 and then centrifuged at 9072 g at 4 °C for 5 min. The supernatant was used for TPC measurement using the Folin–Ciocalteu reagent according to the method described by Singleton and Rossi (1965) [20].

### 2.7. Enzymes Assay: Polyphenol Oxidase (PPO), pHenylalanine Ammonia-Lyase (PAL), Peroxidase (POX), and Superoxide Dismutase (SOD) Activity

For the enzyme extraction, 1 g of hull (pericarp) tissue was homogenized in 10 mL of 50 mM potassium phosphate buffer (pH = 7.2) containing 1% (*w*/*v*) polyvinylpolypyrrolidone (PVP) and 1 mM ethylene diamine tetra acetic acid (EDTA) in falcon tubes (50 mL) using an Ultra-Turrax homogenizer (Janke and Kunkel, Ika-Labortechnik, Germany). The homogenate was centrifuged at 9072 g and 4 °C for 20 min. The supernatant was used to measure the activity of following enzymes. The polyphenol oxidase (PPO) enzyme (EC 1.14.18.1) activity was determined using Jiang’s (1999) [21] method. The phenylalanine ammonia-lyase (PAL) enzyme (EC 4.3.1.24) activity was assayed according to D’Cunha, Satyanarayan, and Nair (1996) [22]. The peroxidase (POX) enzyme (EC 1.11.1.7) activity was assessed by time dependent absorbance changes using guaiacol as a precursor as described by Mohammadi et al. [23]. The superoxide dismutase (SOD) enzyme (EC 1.15.1.1) activity was assessed by 50% inhibition of photochemical-reduction of the nitro-blue-tetrazolium (NBT) according to Giannopolitis and Ries (1977) [24].

### 2.8. Statistical Analysis

The data were analyzed using general linear model (Proc GLM) procedure in SAS 9.4 (SAS Institute, Inc., Cary, NC, USA). The treatment means were statistically compared using least significant differences (LSD, *p* ≤ 0.05). The sources of variation were storage time and the atmospheric treatment combinations, each value was the average of four replications. Prior to all statistical analyses, the residual data were subjected to normality and homogeneity tests of variance. The results were expressed as means ± SE. The Pearson’s correlation coefficient was applied to determine the relationships among the quality parameters studied.

## 3. Results and Discussion

### 3.1. Weight Loss and Microbial Counts

**Figure 1:**
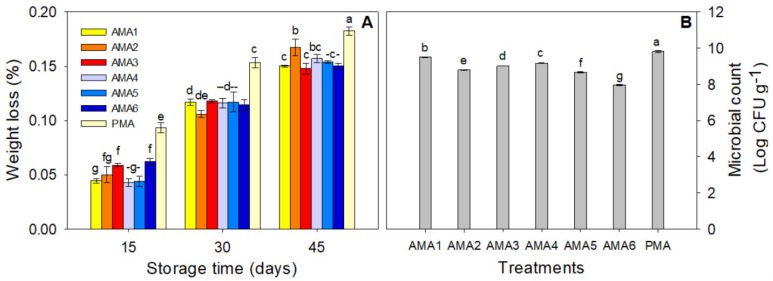
Effect of different active-modified atmosphere packaging (active-MAP) and passive-MAP conditions on weight loss (**A**) through the 45 days of storage, and microbial counts (**B**) at the end of storage of fresh in-hull pistachios stored at 4 ± 1 °C and 90 ± 5% relative humidity (R.H.). Means followed by the same letter for a parameter, are not significantly different by least significant differences (LSD) (*p* ≤ 0.05). Vertical bars indicate the standard errors of four replicates.

Total package weight decreased gradually throughout the storage period. After 45 days of storage the weight loss was significantly higher in the passive-MAP treatment (0.18%) than in the active-MAP treatments (Figure 1A). However, in this study weight loss for all treatments was minimal ranging from 0.04 to 0.18%, demonstrating the significant economic advantage of packaging. Shayanfar, et al. [25] reported less than 0.5% weight loss after 42 days of storage for fresh in-hull pistachios packaged in high barrier polypropylene bags compared to those kept in the open air. Similar results have been reported for fresh hazelnuts under MAP conditions [10]. Microbial counts were evaluated after 45 days of storage. Fruits packaged in active-MAP (AMA)6 and passive-MAP (PMA) had significantly lower (7.96 Log CFU g^−1^) and higher (9.81 Log CFU g^−1^) aerobic mesophilic bacteria counts at the end of storage, respectively, compared to the other treatments (*p* ≤ 0.05, Figure 1B). These results demonstrated decreased aerobic mesophilic bacteria counts with increasing CO_2_/decreasing O_2_ concentrations inside the packages. Gould (1996) [26] reported that elevated CO_2_ concentrations could potentially decrease the microbial growth by 100-fold. The bacteriostatic effect of CO_2_ could be a function of the acidification effect of CO_2_ reducing the pH of growth media, or of decreasing O_2_ availability [27]. Shayanfar et al. [25] and Ozturk et al. [16] reported passive-MAP can significantly decrease microbial counts in fresh in-hull pistachios compared to vacuum or conventional packaging methods.

### 3.2. External Hull Color and Firmness

Among the color parameters, the lightness (L^*^) and a^*^ values of external hull color decreased significantly (*p* ≤ 0.05), and the chroma (C^*^), *h^°^*, and b^*^ values increased significantly as a function of storage time (Table 2). Firmness of packaged pistachios decreased significantly (*p* ≤ 0.05) during the 45 days of cold storage, regardless of the atmospheric combinations, ranging from 1.34–0.70 and 1.14–1.11 N for the hulls and kernels, respectively (Table 2).

### 3.3. Total Chlorophylls and Carotenoids Content

In both kernel and hull tissue of fresh pistachios stored under passive and active-MAP, TCL steadily decreased during 45 days of storage at 4 ± 1 °C (Table 3). The AMA1 had significantly (*p* ≤ 0.05) higher hull TC (11.5 mg kg^−1^) after 15 days of storage. However, by the end of storage no significant differences were observed among the atmospheric combinations. In contrast, fruits stored in the AMA1, AMA2, and AMA3 maintained kernel TCL significantly (*p* ≤ 0.05) better than the other treatments after 45 days of storage. In both kernel and hull tissue of fresh pistachios CAC was significantly (*p* ≤ 0.05) affected by both the passive- and active-MAP treatments showing fluctuations throughout the storage period (Table 3). Maximum CAC of hull tissue was observed in the AMA5 (4.63 mg kg^−1^) after 15 days of storage. These significant differences dissipated after 45 days of storage. In contrast, fruit stored under AMA3 and AMA6 exhibited the highest (9.84 mg kg^−1^) and the lowest (5.83 mg kg^−1^) kernel CAC, respectively, after 45 days of storage (*p* ≤ 0.05). Pistachio is the only nut with chlorophylls in the kernel. Unripe green pistachios have the highest chlorophyll content which then decreases with ripening [28]. The efficacy of modified atmosphere packaging to maintain chlorophylls and carotenoids has been reported for multiple green colored fruit and vegetable including asparagus [29,30], broccoli [31], and chili [32].

### 3.4. Total Anthocyanin Content, Total Antioxidant Activity, and Total Phenolic Compounds

**Figure 2:**
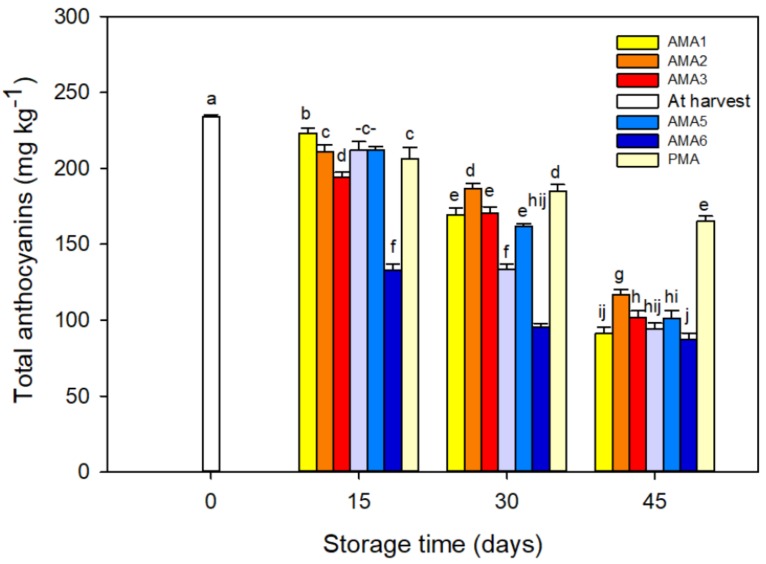
Effect of different active-MAP and passive-MAP conditions on total anthocyanins (TAC) in the kernel’s skin of fresh in-hull pistachios stored for 45 days at 4 ± 1 °C and 90 ± 5% R.H. Means followed by the same letter for a parameter, are not significantly different according to the LSD (*p* ≤ 0.05). Vertical bars indicate the standard errors of four replicates.

The initial value of TAC in the kernel skin (integument) of fresh in-hull pistachios was 234.2 mg kg^−1^ (Figure 2). During storage, the TAC steadily declined, markedly under the AMA6 treatment. By the end of storage, the PMA and AMA6 showed the highest (165.3 mg kg^−1^) and the lowest (87.2 mg kg^−1^) TAC, respectively. The TAC might have been affected negatively by high CO_2_ and low O_2_ levels in the packages, particularly in the AMA6 treatment. Similar results have been reported for pomegranates [33]. A reduction of TAC has been observed in strawberries under high CO_2_ (10%–40%) treatments. This appears to be related to the reduction in activity of uridine diphosphate (UDP): Flavonoid glycosyltransferase (UFGT) enzyme, and a reduction in anthocyanin stability due to changes in pH [34]. The major anthocyanins that generate the red/purple color of pistachio kernel’s integument are cyanidin-3-galactoside and cyanidin-3-glucoside [3,28], and pistachio is the only edible nut containing anthocyanin [35].

The TAA (DPPH radical scavenging activity) in the kernel and hull tissue of the fresh pistachios was significantly (*p* ≤ 0.05) affected by the interaction of atmospheric combinations and storage time (Table 4). By the end of storage, the AMA1 and AMA6 treatments had the highest (91.36%) and the lowest (89.83%) TAA in the hull tissues, respectively. Similarly, by the end of storage, the PMA and AMA5 had the highest (46.11%) and the lowest (38.78%) TAA in the kernel, respectively. Additionally, the hull tissue of fresh pistachios showed an approximately a two-fold higher TAA, than the kernels (Table 4). Throughout 45 days of storage at 4 ± 1 °C the passive- and active-MAP treatments, interacting with storage time, had significant (*p* ≤ 0.05) effects on the kernel and hull tissue TPC of fresh in-hull pistachios (Table 4). After 45 days of storage the PMA (14.15 g kg^−1^ gallic acid equivalent (GAE)) had significantly higher TPC in the hull tissue (*p* ≤ 0.05). However, no significant differences were observed among the other treatments. By the end of storage, the PMA and AMA5 showed the highest (5.36 g kg^−1^ GAE) and the lowest (4.75 g kg^−1^ GAE) TPC in the kernel, respectively. The hull tissue of fresh pistachios had a 2.38-fold higher TPC compared to the kernels (Table 4). In the present study, after 45 days of storage at 4 ± 1 °C, both the passive- and active-MAP treatments maintained the TPC and TAA of fresh pistachio kernel and hull tissue, at the initial or above the harvest levels. Similar results were reported for fresh in-hull walnuts [9], baby spinach [36], and shiitake mushrooms [37] reporting MAP preserved antioxidants and phenolic compounds.

### 3.5. Enzyme Activity

The PPO activity increased steadily in fresh pistachio hulls under all treatments throughout the 45 days of storage at 4 ± 1 °C (Table 5). After 15 days of storage, AMA4 had significantly reduced PPO activity (0.016 U mg^−1^ protein) compared to the other atmospheric combinations (*p* ≤ 0.05). No significant differences were observed in PPO activity after 30 and 45 days of storage (*p* > 0.05). The PAL activity decreased during storage (Table 5). Eventually, AMA1 was the only treatment which showed significantly (*p* ≤ 0.05) higher levels of PAL activity (3.69 U mg^−1^ protein). Triggering PAL activity, a key enzyme that produces the precursor to many protective phenolic compounds through phenylpropanoid pathway, can extend the postharvest life of fruit and vegetables by enhancing the plant’s defense system [38,39]. Activities of the POX enzymes in the hull tissue significantly (*p* ≤ 0.05) increased during the first 15 days of storage, subsequently decreasing sharply and remained at low levels without any significant differences among the treatments through 30 and 45 days of storage (Table 5). The POX enzyme activity is closely related to both antioxidant defense and enzymatic browning [40]. As a protective antioxidant enzyme, POX plays a major role in resisting membrane damage by scavenging reactive oxygen species (ROS) and enzymatically degrading H_2_O_2_ [41,42]. Activities of SOD enzymes in the hull tissue increased gradually from day 0 to day 30, and declined thereafter (Table 5). The SOD is a key enzyme in the plant’s antioxidant defense system, responsible for dismutation of the superoxide (O_2_^−^) radical to O_2_ and H_2_O_2_. Increasing SOD activity in MAP-stored fruit can provide protection against the oxidative membrane damage caused by superoxide radicals [43]. The results given here demonstrate that the active-MAP treatments had a significant effect on maintaining higher SOD enzyme activity. By the end of storage period higher SOD activity was observed in the treatments with the higher CO_2_ levels, the AMA3 (58.39 U mg^−1^ protein), AMA5 (58.75 U mg^−1^ protein), and AMA6 (55.67 U mg^−1^ protein) treatments. Similarly, after 45 days of storage the PMA treatments had the lowest SOD activity (30.07 U mg^−1^ protein). Multiple researchers have reported the positive effects of low O_2_ and high CO_2_ concentrations on enzymatic activity in fresh fruit. Moscetti et al. [10] reported atmospheres lacking oxygen preserved the activity of POX and PPO enzymes at constant levels for 12 days in fresh hazelnut versus the gradual decrease of enzyme activity in air-stored samples. Wang et al. [9] reported MAP treatments increased PAL activity and decreased PPO and POX activity in the green hull of fresh in-hull walnuts stored at −0.5 to 1.0 °C for 60 days.

### 3.6. Correlation Analysis

Table 6 demonstrates the correlations between the color parameters, pigments and PPO activity. The hull TCL had a positive correlation with L^*^ (+0.67, *p* ˂ 0.001) and negative correlation with b^*^ (−0.47, *p* ˂ 0.05). The hull CAC correlated positively with a^*^ (+0.60, *p* ˂ 0.01) and C^*^ (+0.48, *p* ˂ 0.05), and negatively with b^*^ (−0.47, *p* ˂ 0.05) and h° (−0.60, *p* ˂ 0.01). The PPO activity had a positive correlation with b^*^ (+0.64, *p* ˂ 0.001) and h° (+0.71, *p* ˂ 0.001), and a negative correlation with a^*^ (−0.68, *p* ˂ 0.001), C^*^ (−0.47, *p* ˂ 0.05), hull TCL (−0.71, *p* ˂ 0.001), and hull CAC (−0.70, *p* ˂ 0.001). The high TCL in hulls and kernels of pistachio is related to freshness and brightness (high L* value), and it decreases during storage (Table 3) as a result of aging and senescence related chlorophyll degradation [44,45]. As shown in Table 6, the decreased hull TCL and CAC was negatively correlated to increased PPO activity, suggesting it was responsible for the senescence related browning of fresh in-hull pistachios during postharvest storage. The PPO enzyme plays a key role in the enzymatic browning due to de-compartmentalization of enzymes and substrates during senescence and membrane damage in fruits [46,47]. Table 7 shows correlations between the antioxidant substances and total phenolic compounds (TPC). The TAA in the hulls and kernels had a significant positive correlation with the TPC in the hull (+0.51, *p* ˂ 0.05) and kernel (+0.45, *p* ˂ 0.05), respectively (Table 7). Phenolic compounds have a synergistic effect on antioxidant activity [48]. Strong positive correlations have been previously found between TAA and TPC in pistachio [49,50] and walnuts [51,52].

## 4. Conclusions

This is the first report evaluating the potential of active modified atmosphere packaging, using of multiple O_2_, CO_2_, and N_2_ concentrations, to extend the storage life of fresh in-hull pistachios. Two variables, atmospheric combinations and storage time, and their interactions, had significant effects on the physio-chemical parameters of fresh in-hull pistachios during a 45-day storage period. In summary, the different atmospheric treatment combinations had no significant effects on firmness and color parameters. The active-MAP (AMA) treatments were more effective than the passive-MAP (PMA) in preventing weight loss, deterring microbial counts, and maintaining kernel TCL and CAC. The active-MAP treatments promoted higher levels of PAL and SOD enzyme activity than the passive-MAP, while simultaneously preserving the hull and kernel TAA and TPC, levels. Among the active-MAP treatments, the higher CO_2_ treatments, particularly the AMA6 treatment, significantly deterred microbial counts. Considering the significantly affected parameters, weight loss, microbial count, kernel TCL, CAC and TAC, hull and kernel TAA and TPC, and kernel PAL, and SOD, the AMA3 (5% O_2_ + 45% CO_2_ + 50% N_2_) was the optimal gas combination to preserve the postharvest quality of fresh in-hull pistachios. The next step in improving storage of fresh in-hull pistachio packaging is to investigate the effect of low barrier films and micro-perforation techniques, combined with techniques that deter microbial count.

## Figures and Tables

**Table 1:** Experimental factors and different active-modified atmosphere packaging (MAP) (AMA) and passive-MAP (PMA) atmospheric treatment combinations used in this study.

Item	Values/Characteristics		
Factors	A: Atmospheric combination	AMA1 (active-MAP1)	5% O_2_ + 5% CO_2_ + 90% N_2_
		AMA2 (active-MAP2)	5% O_2_ + 25% CO_2_ + 70% N_2_
		AMA3 (active-MAP3)	5% O_2_ + 45% CO_2_ + 50% N_2_
		AMA4 (active-MAP4)	2.5% O_2_ + 5% CO_2_ + 92.5% N_2_
		AMA5 (active-MAP5)	2.5% O_2_ + 25% CO_2_ + 72.5% N_2_
		AMA6 (active-MAP6)	2.5% O_2_ + 45% CO_2_ + 52.5% N_2_
		PMA (passive-MAP)	21% O_2_ + 0.03% CO_2_ + 78% N_2_
	B: Storage time (days)	0 (at harvest), 15, 30, 45	-
Factors’ level	A = 7	Treatments simple effect	-
	B = 4	Storage time simple effect	-
	A × B = 28	Treatment × Storage time interactions	-
Replications	4	-	-
Environmental factors	Storage temperature Relative humidity (R.H.)	4 ± 1 °C 90 ± 5%	- -

**Table 2:** Changes in external hull color parameters (L^*^, a^*^, b^*^, chroma, and *h°*), kernel firmness, and hull firmness of fresh in-hull pistachios packaged under passive- and active-MAP conditions, and stored for 45 days at 4 ± 1 °C and 90 ± 5% R.H.

	Parameters						
Storage Time (Days)	L^*^	a^*^	b^*^	C^*^	*h^°^*	Kernel Firmness (N)	Hull Firmness (N)
0	^x^ 62.14 ± 0.10 a^y^	10.76 ± 0.07 c	16.98 ± 0.09 c	20.11 ± 0.09 c	1.00 ± 0.00 b	1.14 ± 0.003 a	1.34 ± 0.01 a
15	54.58 ± 0.30 b	13.16 ± 0.23 a	17.15 ± 0.16 bc	21.66 ± 0.08 a	0.91 ± 0.01 d	1.13 ± 0.004 b	0.72 ± 0.01 b
30	54.29 ± 0.29 b	12.46 ± 0.21 b	17.54 ± 0.17 ab	21.57 ± 0.08 a	0.95 ± 0.01 c	1.13 ± 0.004 b	0.70 ± 0.01 b
45	54.77 ± 0.25 b	9.99 ± 0.20 d	17.95 ± 0.13 a	20.57 ± 0.07 b	1.06 ± 0.01 a	1.11 ± 0.004 c	0.74 ± 0.01 b

^x^ Data are means ± SE of four replicates. ^y^ Means followed by the same letter, in each column, are not significantly different: LSD (*p* ≤ 0.05).

**Table 3:** Effect of different active-MAP (AMA) and passive-MAP (PMA) conditions on the hull’s total chlorophylls (Hull TCL), kernel’s total chlorophylls (Kernel TCL), hull’s total carotenoids (Hull CAC), and kernel’s total carotenoids (Kernel CAC) of fresh in-hull pistachios stored for 45 days at 4 ± 1 °C and 90 ± 5% R.H.

Parameter	Storage Time (Days)	Treatment						
		AMA1	AMA2	AMA3	AMA4	AMA5	AMA6	PMA
Hull TCL (mg kg^−1^)	0	^x^ 13.68 ± 0.19 a^y^	13.68 ± 0.19 a	13.68 ± 0.19 a	13.68 ± 0.19 a	13.68 ± 0.19 a	13.68 ± 0.19 a	13.68 ± 0.19 a
	15	11.5 ± 0.09 b	9.08 ± 0.16 c	7.14 ± 0.07 d	6.82 ± 0.19 de	5.09 ± 0.13 h	4.92 ± 0.14 h	7.00 ± 0.07 de
	30	5.58 ± 0.10 fg	5.75 ± 0.14 f	5.66 ± 0.12 fg	5.72 ± 0.16 f	5.29 ± 0.15 gh	5.02 ± 0.23 h	6.6 ± 0.11 e
	45	4.48 ± 0.21 i	4.35 ± 0.16 i	4.24 ± 0.05 i	4.31 ± 0.16 i	4.36 ± 0.18 i	4.55 ± 0.17 i	4.4 ± 0.16 i
Kernel TCL (mg kg^−1^)	0	11.67 ± 0.09 a	11.67 ± 0.09 a	11.67 ± 0.09 a	11.67 ± 0.09 a	11.67 ± 0.09 a	11.67 ± 0.09 a	11.67 ± 0.09 a
	15	10.72 ± 0.13 c	11.50 ± 0.17 ab	11.25 ± 0.09 b	9.80 ± 0.16 d	7.38 ± 0.15 e	5.10 ± 0.15 h	11.42 ± 0.10 ab
	30	9.58 ± 0.12 d	7.52 ± 0.10 e	6.76 ± 0.15 f	5.84 ± 0.14 g	4.94 ± 0.21 h	3.66 ± 0.09 jk	7.41 ± 0.09 e
	45	5.00 ± 0.09 h	5.10 ± 0.13 h	5.00 ± 0.08 h	3.96 ± 0.17 ij	3.08 ± 0.16 l	3.44 ± 0.07 kl	4.24 ± 0.18 i
Hull CAC (mg kg^−1^)	0	4.07 ± 0.06 cde	4.07 ± 0.06 cde	4.07 ± 0.06 cde	4.07 ± 0.06 cde	4.07 ± 0.06 cde	4.07 ± 0.06 cde	4.07 ± 0.06 cde
	15	4.18 ± 0.02 bc	3.62 ± 0.03 k	4.02 ± 0.04def	4.11 ± 0.04 cde	4.63 ± 0.07 a	4.10 ± 0.07 cde	4.31 ± 0.06 b
	30	4.16 ± 0.03 cd	4.02 ± 0.05 def	3.78 ± 0.04 hij	3.87 ± 0.05 ghi	3.69 ± 0.02 jk	4.00 ± 0.03 efg	3.90 ± 0.07 fgh
	45	3.71 ± 0.05 jk	3.74 ± 0.04 ijk	3.68 ± 0.04 jk	3.60 ± 0.07 k	3.61 ± 0.05 k	3.66 ± 0.03 jk	3.68 ± 0.04 jk
Kernel CAC (mg kg^−1^)	0	8.4195 ± 0.03 cd	8.4195 ± 0.03 cd	8.4195 ± 0.03 cd	8.4195 ± 0.03 cd	8.4195 ± 0.03 cd	8.4195 ± 0.03 cd	8.4195 ± 0.03 cd
	15	9.0498 ± 0.1 b	8.3588 ± 0.03 cde	9.1703 ± 0.03 b	9.2253 ± 0.11 b	8.1876 ± 0.07 e	7.2076 ± 0.05 g	9.1426 ± 0.07 b
	30	5.8312 ± 0.10 j	7.3809 ± 0.08 fg	6.1495 ± 0.06 i	6.4947 ± 0.13 h	5.8764 ± 0.05 j	8.417 ± 0.1 cd	8.3192 ± 0.09 de
	45	9.0909 ± 0.06 b	7.1607 ± 0.11 g	9.8496 ± 0.05 a	9.2305 ± 0.10 b	7.5551 ± 0.11 f	5.83 ± 0.10 j	8.5699 ± 0.12 c

^x^ Data are presented as means ± SE of four replicates. ^y^ Means followed by the same letter in columns and rows for each parameter are not significantly different: LSD (*p* ≤ 0.05).

**Table 4:** Effect of different AMA and PMA conditions on the hull’s total antioxidant activity (Hull TAA), kernel’s total antioxidant activity (Kernel TAA), hull’s total phenolic compounds (Hull TPC), and kernel’s total phenolic compounds (Kernel TPC) of fresh in-hull pistachios stored for 45 days at 4 ± 1 °C and 90 ± 5% R.H.

Parameter	Storage Time (Days)	Treatment						
		AMA1	AMA2	AMA3	AMA4	AMA5	AMA6	PMA
Hull TAA (%)	0	^x^ 90.19 ± 0.24 ghij^y^	90.19 ± 0.24 ghij	90.19 ± 0.24 ghij	90.19 ± 0.24 ghij	90.19 ± 0.24 ghij	90.19 ± 0.24 ghij	90.19 ± 0.24 ghij
	15	93.69 ± 0.14 a	93.26 ± 0.28 ab	93.1 ± 0.25 ab	90.35 ± 0.29 efghij	90.01±0.32 hij	90.16±0.39 ghij	92.63 ± 0.57 bc
	30	89.4 ± 0.45 j	90.05 ± 0.29 ghij	92.07 ± 0.56 cd	90.91 ± 0.40 efgh	90.13 ± 0.36 ghij	90.21 ± 0.57 ghij	91.27 ± 0.54 def
	45	91.36 ± 0.32 de	91.32 ± 0.31 def	90.33 ± 0.27 fghij	90.45 ± 0.42 efghi	90.65 ± 0.42 efghi	89.83 ± 0.31 ij	91.04 ± 0.25 efg
Kernel TAA (%)	0	44.08 ± 0.92 cd	44.08 ± 0.92 cd	44.08 ± 0.92 cd	44.08 ± 0.92 cd	44.08 ± 0.92cd	44.08 ± 0.92 cd	44.08 ± 0.92 cd
	15	48.90 ± 0.57 a	41.71 ± 0.76efg	40.51 ± 0.97 fghi	40.85 ± 1.16 efghi	38.95 ± 0.77 i	42.50 ± 0.46 def	39.55 ± 0.86 ghi
	30	40.65 ± 1.24 fghi	43.03 ± 0.87 de	39.44 ± 0.78 ghi	40.84 ± 0.33 efghi	41.59 ± 1.22 efg	47.34 ± 0.61 ab	41.00 ± 0.58 efghi
	45	44.37 ± 0.36 cd	41.41 ± 0.77efg	44.15 ± 0.38 cd	39.06 ± 0.55 hi	38.78 ± 1.20 i	41.38 ± 0.38 efgh	46.11 ± 0.66 bc
Hull TPC (g kg^−1^ GAE)	0	11.22 ± 0.38 g	11.22 ± 0.38 g	11.22 ± 0.38 g	11.22 ± 0.38 g	11.22 ± 0.38 g	11.22 ± 0.38 g	11.22 ± 0.38 g
	15	12.60 ± 0.19 ef	15.03 ± 0.62 ab	14.32 ± 0.74 bc	12.88 ± 0.54 def	12.70 ± 0.15 ef	12.96 ± 0.76 def	13.77 ± 0.55 bcde
	30	13.17 ± 0.44 cdef	13.25 ± 0.33 cdef	16.25 ± 0.70 a	12.06 ± 0.30 fg	13.12 ± 0.18 cdef	12.77 ± 0.52 ef	12.87 ± 0.47 def
	45	12.86 ± 0.69 def	12.97 ± 0.40 cdef	12.54 ± 0.51 efg	12.5 ± 0.43 efg	11.92 ± 0.56 fg	12.20 ± 0.21 fg	14.15 ± 0.63 bcd
Kernel TPC (g kg^−1^ GAE)	0	4.71 ± 0.06 gh	4.71 ± 0.06 gh	4.71 ± 0.06 gh	4.71 ± 0.06 gh	4.71 ± 0.06 gh	4.71 ± 0.06 gh	4.71 ± 0.06 gh
	15	4.60 ± 0.10 hi	4.61 ± 0.11 hi	4.56 ± 0.07 hi	4.46 ± 14 ij	4.28 ± 0.03 j	4.47 ± 0.11 ij	4.30 ± 0.9 j
	30	4.95 ± 0.11 def	4.86 ± 0.05 efg	4.58 ± 0.03 hi	5.14 ± 0.04 bcd	4.89 ± 0.06 efg	5.84 ± 0.05 a	5.04 ± 0.03 cde
	45	5.01 ± 0.05 cde	4.92 ± 0.07 defg	5.23 ± 0.11 bc	5.02 ± 0.09 cde	4.75 ± 0.06 fgh	5.27 ± 0.07 b	5.36 ± 0.10 b

^x^ Data are presented as means ± SE of four replicates. ^y^ Means followed by the same letter in columns and rows for each parameter are not significantly different: LSD (*p* ≤ 0.05).

**Table 5:** Effect of different AMA and PMA conditions on the polyphenol oxidase (PPO), phenylalanine ammonia-lyase (PAL), peroxidase (POX), and superoxide dismutase (SOD) activity in the hulls of fresh pistachios stored for 45 days at 4 ± 1 °C and 90 ± 5% R.H.

Parameter	Storage Time (Days)	Treatment						
		AMA1	AMA2	AMA3	AMA4	AMA5	AMA6	PMA
PPO (U mg^−1^ protein)	0	^x^ 0.013 ± 0.00 h^y^	0.013 ± 0.00 h	0.013 ± 0.00 h	0.013 ± 0.00 h	0.013 ± 0.00 h	0.013 ± 0.00 h	0.013 ± 0.00 h
	15	0.025 ± 0.00 e	0.027 ± 0.00 cd	0.026 ± 0.00 de	0.016 ± 0.00 g	0.022 ± 0.00 f	0.026 ± 0.00 de	0.029 ± 0.00 c
	30	0.032 ± 0.00 b	0.032 ± 0.00 b	0.033 ± 0.00 b	0.031 ± 0.00 b	0.032 ± 0.00 b	0.032 ± 0.00 b	0.032 ± 0.00 b
	45	0.044 ± 0.00 a	0.046 ± 0.00 a	0.044 ± 0.00 a	0.045 ± 0.00 a	0.044 ± 0.00 a	0.045 ± 0.00 a	0.044 ± 0.00 a
PAL (U mg^−1^ protein)	0	5.31 ± 0.19 a	5.31 ± 0.19 a	5.31 ± 0.19 a	5.31 ± 0.19 a	5.31 ± 0.19 a	5.31±0.19 a	5.31 ± 0.19 a
	15	4.00 ± 0.13 bc	2.82 ± 0.21 hijkl	3.88 ± 0.36 bcd	4.08 ± 0.23 b	2.96 ± 0.18 ghijk	2.78 ± 0.16 hijkl	2.53 ± 0.08 jkl
	30	3.51 ± 0.19 cdef	2.61 ± 0.08 ijkl	3.22 ± 0.17 efgh	2.82 ± 0.06 hijkl	2.47 ± 0.22 kl	3.03 ± 0.26 fghij	2.43 ± 0.07 l
	45	3.69 ± 0.20 bcde	3.08 ± 0.25 fghi	3.16 ± 0.05 fgh	3.01 ± 0.03 fghij	3.44 ± 0.07 defg	3.37 ± 0.08 efg	3.33 ± 0.06 efg
POX (U mg^−1^ protein)	0	0.008 ± 0.00 d	0.008 ± 0.00 d	0.008 ± 0.00 d	0.008 ± 0.00 d	0.008 ± 0.00 d	0.008 ± 0.00 d	0.008 ± 0.00 d
	15	0.013 ± 0.00 b	0.013 ± 0.00 b	0.011 ± 0.00 c	0.015 ± 0.00 a	0.010 ± 0.00 c	0.011 ± 0.00 c	0.010 ± 0.00 c
	30	0.003 ± 0.00 e	0.003 ± 0.00 e	0.003 ± 0.00 e	0.003 ± 0.00 e	0.002 ± 0.00 e	0.003 ± 0.00 e	0.003 ± 0.00 e
	45	0.002 ± 0.00 e	0.002 ± 0.00 e	0.002 ± 0.00 e	0.002 ± 0.00 e	0.002 ± 0.00 e	0.002 ± 0.00 e	0.002 ± 0.00 e
SOD (U mg^−1^ protein)	0	44.39 ± 0.83 kl	44.39 ± 0.83 kl	44.39 ± 0.83 kl	44.39 ± 0.83 kl	44.39 ± 0.83 kl	44.39 ± 0.83 kl	44.39 ± 0.83 kl
	15	47.07 ± 0.77 jk	51.92 ± 1.06g hi	47.78 ± 1.03 ijk	60.28 ± 1.67 de	66.57 ± 1.07 bc	60.46 ± 1.79 de	52.57 ± 1.88 gh
	30	58.32 ± 2.13 ef	60.10 ± 1.35 de	63.85 ± 1.64 cd	65.78 ± 1.43 c	73.53 ± 1.32 a	70.21 ± 1.20 ab	66.85 ± 0.95 bc
	45	49.71 ± 2.00 hij	37.96 ± 1.13 m	58.39 ± 3.50 ef	41.75 ± 1.16 lm	58.75 ± 1.62 ef	55.67 ± 2.50 fg	30.07 ± 0.76 n

^x^ Data are presented as means ± SE of four replicates. ^y^ Means followed by the same letter in columns and rows for each parameter are not significantly different: LSD (*p* ≤ 0.05).

**Table 6:** Correlation coefficients between external hull color parameters (L^*^, a^*^, b^*^, C^*^, *h^°^*), hull total chlorophylls (hull TCL) and carotenoids content (hull CAC), and PPO activity in the hull tissue of fresh in-hull pistachios stored for 45 days at 4 ± 1 °C and 90 ± 5% R.H.

	L^*^	a^*^	b^*^	C^*^	*h^°^*	Hull TCL	Hull CAC
a^*^	−0.30 ^ns^						
b^*^	−0.02 ^ns^	−0.73 ***					
C^*^	−0.46 *	0.85 ***	0.27 ^ns^				
*h^°^*	0.25 ^ns^	−0.99 ***	0.81 ***	−0.77 ***			
Hull TCL	0.67 ***	0.26 ^ns^	−0.47 *	0.00 ^ns^	−0.32 ^ns^		
Hull CAC	0.13 ^ns^	0.60 **	−0.47 *	0.48 *	−0.60 **	0.31 ^ns^	
PPO	−0.36 ^ns^	−0.68 ***	0.64 ***	−0.47 *	0.71 ***	−0.71 ***	−0.70 ***

*, **, ***, and ns: Significant correlation at the 0.05, 0.01, 0.001 level, and non-significant, respectively. −, negative correlation, otherwise positive correlation.

**Table 7:** Correlation coefficients between TAC, hull TAA and hull TPC, kernel TAA and kernel TPC, PPO, PAL, POX, and SOD activity of fresh in-hull pistachios stored for 45 days at 4 ± 1 °C and 90 ± 5% R.H.

	TAC	Hull TAA	Kernel TAA	Hull TPC	Kernel TPC	PPO	PAL	POX
Hull TAA	0.38 ^ns^							
Kernel TAA	0.00 ^ns^	0.13 ^ns^						
Hull TPC	0.25 ^ns^	0.51 *	−0.15 ^ns^					
Kernel TPC	−0.64 ***	−0.35 ^ns^	0.45 *	−0.23 ^ns^				
PPO	−0.81 ***	−0.12 ^ns^	−0.04 ^ns^	0.00 ^ns^	0.55 **			
PAL	0.25 ^ns^	0.03 ^ns^	0.28 ^ns^	−0.29 ^ns^	−0.11 ^ns^	−0.34 ^ns^		
POX	0.70 ***	0.45 *	0.02 ^ns^	0.13 ^ns^	−0.72 ***	−0.77 ***	0.28 ^ns^	
SOD	−0.03 ^ns^	−0.32 ^ns^	−0.22 ^ns^	−0.04 ^ns^	0.00 ^ns^	−0.27 ^ns^	−0.41 ^ns^	−0.04 ^ns^

*, **, ***, and ns: Significant correlation at the 0.05, 0.01, 0.001 level, and non-significant, respectively. −, negative correlation, otherwise positive correlation.
